# The Challenges of Advance Care Planning for Acute Care Registered Nurses

**DOI:** 10.1177/08445621241244532

**Published:** 2024-04-04

**Authors:** Lori L. Rietze, Kelli I. Stajduhar, Mary Ellen Purkis, Denise Cloutier

**Affiliations:** 1 School of Nursing, 7728 Laurentian University, Sudbury, ON, Canada; 2 School of Nursing, 8205 University of Victoria, Canada; 3 8205 University of Victoria, Canada

**Keywords:** advance care planning, acute care nursing staff, palliative care, end-of-life care, Canada

## Abstract

**Study Background:**

The practice of acute care nurses is shaped by organizational factors such as lack of privacy, heavy workloads, unclear roles, lack of time, and lack of specific policies and procedures. We know little about the social and organizational structures and processes that influence nurses’ uptake of valuable patient-centered discussions like advance care planning (ACP). ACP is beneficial for patients, their substitute decision makers, and healthcare providers.

**Purpose:**

To describe the operational, organizational, and societal influences shaping nurses’ ACP work in acute care settings.

**Methods:**

This ethnographic study purposively sampled 14 registered nurses and 9 administrators who worked in two acute care hospitals in Northeastern Ontario. Methods consisted of 23 open-ended, semi-structured interviews, 20 hours of observational fieldwork, and a collection of publicly available organizational documents. Data were inductively analyzed using an iterative constant comparative approach.

**Results:**

Nurses were challenged to meet multiple competing demands, leaving them to scramble to manage complex and critically ill acute care patients while also fulfilling organizational tasks aligned with funding metrics, accreditation, and strategic planning priorities. Such factors limited nurses’ capacity to engage their patients in ACP.

**Conclusions:**

Acute care settings that align patient values and medical treatment need to foster ACP practices by revising organizational policies and processes to support this outcome, analyzing the tasks of healthcare providers to determine who might best address it, and budgeting how to support it with additional resources.

## Advance care planning for acute care registered nurses: an unattainable goal

### Background

It is estimated that by 2041, 25% of the Canadian population will be 65 years of age or older (Statistics Canada, 2022). Experts associate this trend with advancements in medical research and technology, widespread lifestyle changes such as smoking cessation, healthier diets, increased physical activity, and environmental changes (Statistics Canada, 2019). While these changes have made Canadians healthier, increasing life expectancies means that more older adults live with multiple comorbidities that often require admission to acute care settings for chronic illnesses or for end-of-life care (CIHI, 2021; Miedema, 2013). However, not all acute care experiences by older patients at end-of-life are positive (Heckel et al., 2020). Some patients report that they received treatments that were not consistent with their end-of-life wishes (Drought & Koenig, 2002; Heyland et al., 2013; Teno et al., 2000). This is not a new issue. Dating back to 1995, Connors et al. (1995) found that almost half of the patients at end-of-life were receiving aggressive treatments. The trend continued in 2011, where the majority of healthcare costs at end-of-life were spent on aggressive acute care treatments (Walker et al., 2011). In addition to overtreatment, hospitalized patients at end-of-life also report undertreatment (Lorenc et al., 2021; Mirarchi & Mason Pope, 2023), with unrelieved physical symptoms such as pain (Clark et al., 2015) and unaddressed psychological needs (Gagnon & Duggleby, 2014).

The challenges of dying in acute care settings have led to several initiatives to improve the quality of patient care. Some of these initiatives are the development of inpatient palliative care teams (Higginson et al., 2002), standardized frameworks and pathways to guide care (Gold Standards Framework, 2010), educational initiatives (Shorr et al., 2000), and communication programs (DeCourcey et al., 2021). Extensive attention has also been given to a participatory upstream communication process called advance care planning (ACP), aimed at improving the dying experience for patients and their families.

Researchers found that ACP is beneficial for patients, their substitute decision makers (SDMs), and healthcare providers. Most importantly, ACP discussions at end-of-life have been found to increase the likelihood that healthcare treatment will be consistent with the patient's values (Brinkman-Stoppelenburg et al., 2014; Houben et al., 2014) leading to increased quality of end-of-life care and patient control, improved end-of-life communication, reduced decision-making burden for family members, and reduced moral distress for staff (Brinkman-Stoppelenburg et al., 2014; Detering et al., 2010; Houben et al., 2014; Jimenez et al., 2018; McMahan et al., 2020; Stewart et al., 2011). There are also economic benefits to ACP conversations in that they lead to reduced hospital admissions and lower healthcare costs overall (Starr et al., 2019).

While ACP can have positive effects on patients and SDMs, less than 50% of the general population has engaged in ACP (Canadian Hospice Palliative Care Association, 2013). One of the reasons for low uptake of ACP by Canadians may be the relative lack of clarity on who should be responsible for initiating ACP conversations and in which healthcare settings.

Some scholars argue that since nurses are the patient's most consistent healthcare provider within acute care settings, they are in a unique position to engage hospitalized patients with life-limiting illnesses in conversations to clarify their values and fears with their SDMs (Cadge et al., 2021; Izumi, 2017). However, ACP is still not a widespread role taken by acute care nurses. In North America, few nurses engage their acute care patients in ACP. In Canada, nurses report engaging 20–40% of their patient population in ACP (Boyd et al., 2011; Duke & Thompson, 2007; Rietze et al., 2018); while in the US the figure is similar at about 31% (Arnett et al., 2016). From a patient perspective, even fewer patients report having had ACP conversations with their nurses. Studies of Canadian (Heyland et al., 2013) and Australian (Detering et al., 2021) patients indicate that only 5.8% to 8% recall having had an ACP discussion with their registered nurse. The discrepancy between these numbers may be related to over-reporting by nurses and/or under-reporting by patients.

There are diverse reasons for low engagement in ACP by acute care nurses including heavy workloads that do not allow for uninterrupted time in a private setting to engage in ACP conversations with patients and SDMs (Rietze & Stajduhar, 2015; Rietze et al., 2018; Schulman-Green et al., 2005). These studies found that nurses typically spend only ¼ of their shift in direct patient care with their patients, resulting in little time for engaging in ACP (Allen, 2014, 2015; Hobgood et al., 2005). From an institutional standpoint, most acute care organizations lack policies and procedures related to who should engage in ACP with patients, when it should take place, and if it is essential in the care of patients (Baughman et al., 2015; Detering et al., 2021; Martina et al., 2021; Mohan et al., 2020; Rietze et al., 2018). Consequently, nurses are not clear whether ACP is a part of their scope of practice in acute care settings (Putman-Casdorph et al., 2009; Rietze et al., 2018; Vanderhaeghen et al., 2018) since they receive limited direction, education, training, and templates on how to engage patients in ACP (Baughman et al., 2015; Martina et al., 2021; Whitehead et al., 2021), and are uncertain how, or if, ACP influences treatment planning in their organization (Duke & Thompson, 2007; Putman-Casdorph et al., 2009).

Current research provides a good foundation to appreciate the context in which nurses are making practice decisions about ACP with patients, but “the current healthcare system…[is] notoriously resistant to change” (Thorne & Stajduhar, 2017, p. 2). It is this resiliency that underscores the importance for “a comprehensive understanding of the kinds of cultural and attitudinal factors” (Thorne & Stajduhar, 2017, p. 2) that influence acute care settings. Similarly, we have a limited understanding of how larger contextual, social, and organizational factors in acute care institutions might influence the capacity of nurses to take up an ACP role (Gagnon & Duggleby, 2014; Scott et al., 2003, p. 926). Advancing ACP in acute care settings must be done with a comprehensive understanding of the culture, discourses, competing interests, and political or public policies that impact nurses’ work in the hospital setting. There remains a gap in knowledge on how operational, organizational, and societal factors might have an impact on nurses’ engagement in ACP practice in hospital settings.

### Purpose

The purpose of this study was to describe the operational, organizational, and societal influences that shape the ACP work of nurses. The research question guiding this study was: How is nursing practice shaped and influenced by the organizational context of acute care, to enable or constrain nurses’ efforts in ACP?

## Methods and procedures

### Study design

The design of this study was classical ethnography with the end goal of describing the organizational context as a social phenomenon, and the decision-making process of its people within their context of everyday work (Clifford & Marcus, 1986; Denzin & Lincoln, 2011; Geertz, 1973). In this study, we were interested in understanding how organizational contexts shaped nurses’ everyday practice and ethnography was the chosen methodology to enable us to use multiple methods to explore the culture of the organizational context as a social phenomenon, and the decision-making process of its people within their context (Denzin & Lincoln, 2011).

### Setting and sample

The setting of the study took place on medical, cardiology, and palliative/oncology units located within two acute care hospitals in Northeastern Ontario. The typical case purposive sample for this study was English-speaking registered nurses (*n* = 14) who worked on these units and organizational administrators (*n* = 9) who oversaw the operation of the study unit such as unit managers or members of the senior management team. We used this sampling method to illustrate the typical patterns and processes across the acute care setting and we aimed for an ethnographic sample size of 20–30 in accordance with Guest et al. (2017). Participants were recruited on the basis of recommendations from the unit managers using the following strategies: 1) *big net approach* where LR interacted with the whole target population in the study setting to build rapport (Fetterman, 1998, p. 32), 2) LR emailed the study poster to target population, 3) the study poster was displayed on the unit, 4) a text number and QR code on poster enabled researcher contact, and 5) LR offered flexible data collection times and formats.

### Protection of human subjects

Ethical approval for the study was obtained from the University of Victoria Human Research Ethics Board, the Research Ethics Board of Laurentian University, and Research Ethics Boards of the primary and secondary research settings.

### Data collection

In accordance with ethnography, multiple distinct data sources were used to collect data (Creswell & Poth, 2018). The main method for collecting data was observation and we used this method to acquire a sense of nurses’ work practices relative to ACP, and learn the social structure and functioning of the unit. Observational data came from LR's presence in the nursing station, attending unit rounds, shadowing registered nurses as they worked, and attending meetings. No observations took place in patient rooms. LR completed 20 hours of observation with insights recorded using a field note book, reflexive journaling, and audio notes. Field notes and memos were written based on observational experiences. With the use of a piloted interview guide, LR conducted 23 semi-structured open-ended interviews with participants in person or online. We also collected publicly available documents to supplement the primary data.

### Data analysis and interpretation

The primary inductive thematic analysis of transcribed field notes, interview transcripts, and documents was completed by LR and KS with support from DC and MEP. All authors then met and used the constant comparative method to collaboratively create a composite description of the social organization of nursing practice within acute care organizations. This data arises from the PhD research of LR and her supervisor and dissertation committee. LR and KS are nurses who have worked in acute and community-based palliative care settings. MEP is a nurse, who has worked in acute care and has conducted research in home care settings. DC is a social gerontologist and health geographer, who does community-engaged research on the care of older adult populations. In this ethnographic study, the process of data collection and data analysis was iterative in that collection and analysis were undertaken concurrently (Hammersley & Atkinson, 2007). During the iterative process, constant comparison of the existing data and subsequent areas for data collection allowed for flexibility in the data collection to expand on emerging concepts, and enabled the researchers to ask more detailed questions during subsequent rounds of collection (Carter & Little, 2007). To assist in data analysis, NVivo (version 13) was used for the purpose of categorizing, organizing, and retrieving data during the process of collapsing or comparing codes. Publicly available documents such as strategic plans, mission statements, and accreditation reports were used to supplement our primary interview and observational data. For instance, when a document was mentioned in the interview, we retrieved it if it was publicly available. After closely reading each document, LR, supported by KS, MEP, and DC constructed themes related to nurses’ roles in ACP, end-of-life care discussions, and nurses’ work more generally. We then compared the themes with the existing data analysis (Morgan, 2022). As a whole, trustworthiness of the data was ensured by methodological coherence. There was consistency between the purpose of the study (to describe influences on behavior within a culture), methodology (ethnography), and methods (interviews, participant observation, and documents) (Carter & Little, 2007). We also triangulated observational field notes, interviews, memos, and documents (Creswell & Poth, 2018), collaboratively analyzed the raw data among all of the researchers, and thickly described the data within the themes (Lincoln & Guba, 1985).

## Results

Nurse participants ranged in age from 26 to 54 years of age and the majority (57.1%) had an undergraduate degree or a diploma (35.7%). Most nurses did not have additional training in palliative care (71.4%) although all but one participant had at least one patient in the previous week who was at end-of-life. Of the administrators, ages ranged from 52 to 60 years and the highest level of education was equally split at 33.3% for each of the diploma, undergraduate degree, and graduate degree categories. See Table 1 for the demographic characteristics of the participants.

**Table 1.** Sample demographics.

	Administrators (*n* = 9)	Registered Nurses (*n* = 14)
	*Mean (range)*	*Mean (range)*
Age, year	55.8 (52-60)	41.1 (26-54)
Number of years worked in organization	24.9 (1-33)	12.5 (2–27)
	*n (%)*	*n (%)*
Sex		
Male	1 (11.1)	1 (7.1)
Female	8 (88.9)	13 (92.9)
Other	0 (0.0)	0 (0.0)
Highest level of education completed		
Diploma	3 (33.3)	5 (35.7)
Undergraduate degree	3 (33.3)	8 (57.1)
Graduate degree	3 (33.3)	1 (7.1)
Discipline of formal training		
Nursing	8 (88.9)	*
Other	1 (11.1)	*
Number of years on unit		
0–3	2 (22.2)	3 (21.4)
4–7	3 (33.3)	4 (28.6)
8–11	2 (22.2)	2 (14.3)
12+	2 (22.2)	5 (35.7)
Additional courses in palliative care		
Yes		4 (28.6)
No		10 (71.4)
Use of ACP in personal life (outside of their employment)		
Yes	*	7 (50.0)
No	*	7 (50.0)
Estimated number of patients/week at EOL		
0	*	1 (7.1)
1–2	*	8 (57.1)
3–4	*	5 (35.7)
4+		0 (0.0)
Typical shift worked		
Days	*	4 (28.6)
Evenings	*	0 (0.0)
Nights	*	0 (0.0)
Mixture	*	10 (71.4)

* not applicable.

ACP, advance care planning; EOL, End-of-life.

### Themes

Four main themes arose from this study: 1) multiple competing organizational demands, 2) “putting out fires,” 3) workforce issues, and 4) the undervaluing of nurses.

### Multiple competing organizational demands

Acute care work environments within which participants practiced were characterized by various competing organizational demands. Most nurses were overwhelmed and frustrated with the organizational imposition of these tasks that competed for their time to care for patients. Managing multiple competing demands during their shift left nurses feeling frantic, unsettled, and preparing to leave their job. One of the oncology nurses said: “it takes a toll on me…I can’t wait to get out of here [acute care hospital nursing].” Another nurse from the cardiology unit indicated that the work is so overwhelming that she discourages younger people from going into nursing.

Participants spoke of organizational pressures to prioritize tasks aligned with organizational values, goals, and strategic directions. Nurses spoke about organizational pressures to prioritize non-patient tasks like medication reconciliation and monthly self-learning plans in order to meet accreditation requirements, which increased their workloads. When asked about ACP, a manager from a medical unit commented, “I don’t think it [ACP] has gone away, I think it has just gotten superseded by other more important, strategic objectives.” Another competing demand that participants spoke of was achieving benchmarks in funding-related metrics such as the efficient patient flow of admissions and discharges. One senior administrator described the organizational focus on performance metrics associated with funding such as “length of stay…and time to in-patient bed”. While many nurses understood the organizational focus on shorter lengths of stay and earlier discharges, they struggled to meet those organizational benchmarks. For instance, one cardiology nurse said: “we always have the pressure to hurry up, get them moving, get them out. I cannot work any faster to get these patients in and out,” while another nurse working on the medical unit agreed, adding: “we cannot work any faster, we need more help, like we need more front-line workers to help with the patients that we do have, and no one seems to be listening.” Working in this context, nurses and administrator participants admitted that ACP conversations, while important, were not an organizational priority in the everyday work of acute care nurses. Since ACP was not a benchmarked metric expected in any of the organizations, there were few organizational processes, structures, motivations, or resources to support its implementation, and nurses did not initiate it with patients. Even in situations where perhaps a patient may have benefited from ACP, nurse participants felt that they had little support or guidance to engage in such a patient-centered process. Rather than considering ACP as an embedded part of nurses’ patient-centered care, participants spoke of it as a distinct task they would need to add to their existing workload if they had time.

### ‘Putting out fires’

From an operational perspective, nurse participants spoke of the lack of time they had to provide care for each of their patients from the very beginning. Nursing work in acute care was often characterized by participants as *putting out fires* as they responded to unpredictably changing workloads, mentoring new nurses, and juggling various complex patient needs. Many nurses used the analogy of *battling against fire* to describe their workloads. Interviews with nurses and insights from observational fieldwork suggested that nurses were scrambling to manage the routine care of patients with complex care plans all under the threat of their patient assignment changing at any moment. One cardiology nurse described how overwhelmed she was in having such a heavy workload: “you just have to wing it sometimes.” In describing the realities of acute care nursing practice, they expressed routinely having to juggle the medical care of complex critically ill patients with alternative-level-of-care patients (ALC), adjust to shifting clinical assignments and re-assignment to other units, and mentor new/inexperienced nurses. The constant re-prioritization of work to address routine and spontaneously arising demands meant that many nurses had to miss their breaks in order to get their work done. One unit manager recognized the insurmountable challenges nurses experienced trying to engage patients in ACP on the oncology unit when they previously attempted it, “It soon became impossible because of the turnover of this floor.” As one medical nurse said:

It's like we are jumping from fire to fire putting each one out until the end of our shift. We are just surviving each little crisis in the midst of overcapacity and understaffing.

Another nurse who worked on the oncology unit described the unpredictability of her work and how the busyness of attending to multiple demands left little time for personalized care of patients:

It's more like spot care - kinda putting out fires rather than tending to their needs or the needs of the family. We are just so busy. Our environment is dynamic and unpredictable, it seems like we are just putting out fires and not really having the time to spend one on one with anybody. I do sit and talk to them, but not like I used to, like, those things don’t worry me as much anymore, like it's more like did the task get done? And did it get done properly?

Given this context, several nurse participants described feeling unprepared and unable to talk with their patients about any sensitive and time-consuming subjects, including ACP. One palliative nurse participant stated this clearly, “we don’t have time to talk to patients. I barely have time to look them in the eye once an hour and give them the pills that they are supposed to get on time before I run to the next one.” Having a lack of time in their clinical day due to mentoring colleagues, caring for complex patients, and changing workload assignments resulted in nurses only being able to do the bare minimum for patients. Being unable to do more for patients and having to leave some tasks undone left many nurses distressed that they were not giving patients the best care.

### Workforce issues

Nursing workforce issues added complexity and busyness to nurses’ work. Many experienced nurses commented on the challenges of the nursing workforce today as seen by nurses leaving their jobs, and new graduates being unprepared for what was expected of them. In some cases, junior nursing staff, having only been a nurse for two years, were considered senior in their positions. For some nurses, working with only young, inexperienced nurses left them feeling nervous that patient care was at risk and that they would not be able to adequately respond to an emergency on their unit. Other nurse participants were concerned that junior nurses did not have the kind of mentoring that was necessary to develop critical skills like multi-tasking, prioritization of patient care, and complex relational care. Some participants said that nurses with less experience tended to focus more on the tasks associated with the role, and those that the organization expected of them. An administrator on the medical unit also commented that she noticed that as nursing experience increased, the likelihood of engaging in ACP-type conversations increased too, “some of us older ones definitely might broach the subject - the younger newer practitioners are going to shy away from topics like that”. While many senior nurses understood their junior colleagues’ need to build necessary skills, one palliative care nurse lamented that new nurses were “uncomfortable asking that [ACP] question. Seeing the unit blend with the number of inexperienced staff that we have sometimes, I can see how the question is not routinely initiated on our unit.” An administrator from the cardiology unit agreed that when they piloted ACP on their unit, they did not have adequate staffing to support it, “we didn’t have the available experienced people that we needed…we didn’t have an ACP team that was able to do this”. The mix of nursing experience, or the relative lack of experienced nurses, and the perception that ACP is an advanced communication process, means that ACP was something that was infrequently addressed on these units.

### Undervaluing of nurses

In line with the general business-like model of acute care, nurse participants described that they felt uninvolved (or sometimes even unrecognized or excluded) in organizational decision-making related to unit initiatives, even though they spent the most time doing the business of healthcare. Many of the nurses stated that they were frustrated with the lack of opportunities to be involved in the prioritization of organizational initiatives on their unit, have input into how to best implement strategic directions, or provide evaluative feedback. A nurse working on the cardiology unit stated:

It's just a matter of this needs to be done and you guys are the ones that are going to be doing it. They never asked us if it should be done, or how it might be best adopted. Management just decided it would now be an expectation on our workload.

One oncology nurse described her view of how initiatives are introduced and managed on her unit:

It is just thrown on us with little empathy or even acknowledgment of how this was affecting the management of our existing work with patients. But it seems like this is the way the hospital leads and the way they do stuff. It's just, this is the expectation that new tasks are done and there are no excuses why it can’t get done.

This kind of top-down decision-making was perceived by many of the nurse participants to be a common practice that left them feeling that their expertise in patient-centered care was undervalued and their concerns about patient safety were consistently diminished or dismissed. Their lack of positional power, agency, and voice in organizational decisions left participants feeling disengaged with management, uncommitted to organizational initiatives, and discouraged that initiatives they found valuable to patient care (i.e., ACP) were not part of the direction of the organization. No administrator interviews referred specifically to the valuing of nurses in the healthcare setting.

## Discussion

This study describes how nursing practice is shaped and influenced by the organizational context of acute care to constrain nurses’ efforts in ACP. Participants described that their heavy workloads and organizationally prioritized tasks were insurmountable barriers to engaging in ACP with patients. Although nurse participants stated that they felt ACP conversations were important for patients to have with healthcare providers in acute care settings, they did not have an avenue to provide feedback on or become involved in point-of-care decisions on their unit such as gaining the resources required to support the implementation of ACP.

In relation to previous research, this study provides a contemporary lens on the intricate organizational processes that may result in the deprioritizing of ACP conversations between nurses and their patients in acute care settings. Findings from this study confirm that acute care nurses are not regularly engaging their patients in ACP conversations. Participating nurses spoke of how their workload increased in the wake of understaffing and contextual changes like the addition of mandated learning modules, mentorship of new nurses, unpredictably changing workload assignments, increased patient age, and the concomitant extreme complexity and diversity in the healthcare needs of patients on their unit. In light of their overwhelming busyness, nurses said that it was impossible for them to add ACP to their current workload. Findings from this study corroborate previous studies. It is not new that nurses describe a sense of feeling undervalued in acute care settings, and silenced related to their patient care concerns (Bourgault, 2022; Thorne & Stajduhar, 2017). In part, this could be related to binding confidentiality agreements where they cannot discuss their concerns in wider forums (Lefebre et al., 2020) or the loss of nursing middle management in hospital settings (Thorne & Stajduhar, 2017). Distress in the acute care work environment was a prominent concern for participants. Literature supports that this is important to consider in reducing nurse burnout because there is an inverse relationship between the quality of the acute care work environment and nurse burnout (Brooks Carthon et al., 2021). Many nurses in this study spoke about their exhaustion, being frustrated with their work setting, and feeling panicked, frazzled, and like they were drowning. The feeling of burnout is common in acute care nursing, being reported by one in every three acute care nurses (Brooks Carthon et al., 2021). Some of the sources of their burnout cited in the literature are heavier workloads, increased patient acuity, time pressures, and limited resources (Jun et al., 2021; Maunder et al., 2021). This study corroborated these themes, where participants cited feelings of distress about their inability to care for patients as they felt they should, and having to leave tasks undone.

However, this study found important and novel factors impacting nurses’ ability to engage their patients in ACP regularly that were not previously found or reported in the literature. For instance, a powerful influencer that shapes nurses’ ACP work is the strong undercurrent in acute care dictating the tasks that *must be prioritized* by nurses in pursuit of systematic benchmarks that demonstrate organizational excellence in efficiency, productivity, and patient flow while meeting accreditation standards. In line with this, there was an organizational preoccupation and resourcing to meet business foci such as funding metrics and accreditation priorities while wide patient-centered care and associated relational practices such as ACP were the resource trade-off. The impact of nurse experience on ACP competency was also a new finding. Nurse participants observed that inexperienced nurses exclusively focused on clinical task mastery rather than engaging in relational care like ACP. Within an acute care context of high nurse turn over then, where the staffing mix was largely inexperienced healthcare staff, the staffing environment did not support ACP implementation. Insufficient staff nurses in acute care settings also set the stage for limited ACP because nurses reported spending any available non-clinical time mentoring novice nurses in lieu of ACP.

### Implications

Healthcare administrators are encouraged to consider the health of the acute care workplace and how the organization can better support nurses to reduce burnout and improve job satisfaction. For instance, participants specifically suggested unit administrators consider policies and procedures regarding safe ways that staff can provide feedback related to practice issues, and strategies to better protect and support nurses in their work. Unit managers may also capitalize on opportunities for regular debriefing sessions with staff and conducting meetings to solicit feedback on new initiatives that impact nursing practice. Being more present on site on the unit on a day-to-day basis so that they can better understand and represent clinical issues impacting nurses and their patients would also be meaningful. Findings from this study encourage acute care administrators to recognize that organizational policies and processes flow through them and that these govern the practices of nurses on their unit and the power they have to advocate for needed change. These recommendations for acute care administrators might be useful in addressing the systemic nature of silencing and voicelessness of nurses and it may reduce nurses’ feelings of burnout and distress. In terms of ACP, if organizations aim to increase end-of-life discussions between nurses and their patients, administrators could advocate for the need for long-term, full-time staff members to complete ACP training.

Findings from this study have recommendations for registered nurses working in acute care organizations. Registered nurses might consider becoming involved in coalitions, professional organizations, and unions to promote avenues for nurses to have a voice to advocate for required changes in acute care settings. In terms of ACP, nurses stated in this study that they could not manage to broach the subject with their patients due to competing demands on their time. On one hand, given the context characterizing contemporary acute care organizations, ACP may not be a realistic option for nurses to consider in their work with patients. Perhaps goals of care discussions are most appropriately focused on acute care, with the movement of ACP into primary care settings (Howard et al., 2020). On the other hand, if ACP is discussed in acute care settings, it could be added into the routine nursing care plan of patients with palliative or ALC diagnoses to ensure their end-of-life wishes are consistent with medical treatment. Resources would need to be re-routed to support this on a full-time basis. Nurses might advocate for the worth of ACP for ALC patients with administrators of their acute care settings. Such an initiative could lead to the establishment of ground-breaking policies and procedures to enable ACP processes in acute care.

Nurse educators are encouraged to teach students how to advocate for patient needs and patient-centered care in a system where they might feel that nurses’ voices, experience, and input is not actively or adequately sought by decision-makers. There are also additional implications for nurse educators who teach leadership courses in that student nurses need the skillset to understand the systemic factors that influence funding and evaluation of funds supporting acute care hospitals as well as how it all impacts nursing practice.

Health policy makers are encouraged to reflect on an insight posed by Gordon (2005) almost two decades ago: “*Within the churn for efficiency in managing healthcare money, is there room to more effectively manage care?”* As seen above, there are multiple examples of where participants state that the structure and processes on their units prioritize efficiency and productivity over patient care. Surely, it is self-evident and we can all agree that healthcare resources need to be managed efficiently, but perhaps there needs to continue to be pointed discussions on the most effective way to manage these dollars within the context of foundational concepts like patient-centred care and patient safety. This requires reflecting on how we got to the place where efficiency in healthcare pushed these concepts to the background.

### Limitations

In any research study, it is important to give careful consideration to limitations on the extent to which claims arising from the analysis can be pushed. In the case of this study, we did need to cut our data collection short due to institutional policies implemented at the outset of the COVID-19 pandemic. At the time these policies were implemented, 23 participants had consented to participate in the study. These participants were made up primarily of nurses (*n* = 14) and the remaining participants were managers and administrators (*n* = 9). We had participants from both intended employment groups (e.g., nurses and managers/administrators) who could provide *different* perspectives on practices of caring for hospitalized patients nearing the end-of-life. These different perspectives provided analytic opportunities for us to explore features of the practice and policy context of care that each nurse and each administrator/manager used to generate their accounts of what made it possible for them to engage in ACP and what interfered with their ability to do so. It was these differences we were interested in obtaining during data collection and we triangulated those accounts which underpinned the strength of our analysis.

## Conclusion

Based on the study findings, nurses do not routinely engage their acute care patients in ACP due to a variety of individual, organizational, and societal factors that work in a concerted way to govern the practice of nurses in acute care settings. Two of the most powerful influences that shape nurses’ work are values of efficiency and productivity which also underpin their overwhelming busyness. Consequently, the preoccupation with a business model of healthcare has pushed out patient-centred care and associated relational practice such as ACP, while reinforcing the silencing of nurses.
